# Aqueous Angiography: Real-Time and Physiologic Aqueous Humor Outflow Imaging

**DOI:** 10.1371/journal.pone.0147176

**Published:** 2016-01-25

**Authors:** Sindhu Saraswathy, James C. H. Tan, Fei Yu, Brian A. Francis, David R. Hinton, Robert N. Weinreb, Alex S. Huang

**Affiliations:** 1 Doheny Eye Institute, Los Angeles, California, United States of America; 2 Department of Ophthalmology, David Geffen School of Medicine at UCLA, Los Angeles, California, United States of America; 3 Department of Biostatistics, David Geffen School of Medicine at UCLA, Los Angeles, California, United States of America; 4 Department of Ophthalmology and Pathology, University of Southern California, Los Angeles, California, United States of America; 5 Hamilton Glaucoma Center and Shiley Eye Institute, University of California San Diego, San Diego, California, United States of America; Massachusetts Eye & Ear Infirmary, Harvard Medical School, UNITED STATES

## Abstract

**Purpose:**

Trabecular meshwork (TM) bypass surgeries attempt to enhance aqueous humor outflow (AHO) to lower intraocular pressure (IOP). While TM bypass results are promising, inconsistent success is seen. One hypothesis for this variability rests upon segmental (non-360 degrees uniform) AHO. We describe aqueous angiography as a real-time and physiologic AHO imaging technique in model eyes as a way to simulate live AHO imaging.

**Methods:**

Pig (n = 46) and human (n = 6) enucleated eyes were obtained, orientated based upon inferior oblique insertion, and pre-perfused with balanced salt solution via a Lewicky AC maintainer through a 1mm side-port. Fluorescein (2.5%) was introduced intracamerally at 10 or 30 mm Hg. With an angiographer, infrared and fluorescent (486 nm) images were acquired. Image processing allowed for collection of pixel information based on intensity or location for statistical analyses. Concurrent OCT was performed, and fixable fluorescent dextrans were introduced into the eye for histological analysis of angiographically active areas.

**Results:**

Aqueous angiography yielded high quality images with segmental patterns (*p*<0.0001; Kruskal-Wallis test). No single quadrant was consistently identified as the primary quadrant of angiographic signal (*p* = 0.06–0.86; Kruskal-Wallis test). Regions of high proximal signal did not necessarily correlate with regions of high distal signal. Angiographically positive but not negative areas demonstrated intrascleral lumens on OCT images. Aqueous angiography with fluorescent dextrans led to their trapping in AHO pathways.

**Conclusions:**

Aqueous angiography is a real-time and physiologic AHO imaging technique in model eyes.

## Introduction

The conventional aqueous humor outflow (AHO) pathways in the eye can be divided into proximal and distal portions.[1–5] The proximal portion starts from the anterior chamber and extends past the trabecular meshwork (TM) into Schlemm’s canal (SC) and toward collector channels (CC).[2, 3] The distal portion starts from collector channels and then moves into the small caliber intrascleral venous plexus, joining aqueous veins in series and parallel with the plexus, and finally flowing into episcleral veins [6, 7] carrying the fluid back to the venous circulation.[1–3] Viewed externally, AHO may thus appear to extend radially away from the limbus 360-degrees circumferentially and uniformly.

Rather than being solely uniform, segmental (non-360 degrees uniform) AHO has been described in multiple systems.[1] Introduction of labelled beads into the anterior chamber of the eye has shown segmental localization in the trabecular meshwork.[8–18] Segmentalization can also be altered by drug treatment.[9] Possible physical theories for proximal AHO pathway segmentalization include funneling.[19] Alternatively, channelography or canalography involve unroofing of SC during glaucoma surgery followed by injection of tracers into SC.[20–25] Channelography/canalography, while focused on distal AHO pathways, also demonstrate non-uniform and segmental AHO.

Experience with Minimally Invasive Glaucoma Surgeries (MIGS) suggests that segmental AHO may be clinically relevant.[26] Trabecular bypass MIGS such as iStent (Glaukos Corporation, Laguna Hills, CA) or Trabectome (Neomedix Corporation, Tustin, CA) utilize TM bypass or ablation to lower intraocular pressure (IOP).[27–31] MIGS are generally conducted in the nasal quadrant of the eye from a temporal clear cornea approach. While MIGS can lower IOP, the extent of lowering is variable and not predictable.[27–31] This suggests that AHO in the normal and/or glaucomatous eye may be more complex than previously understood. Something either in AHO pathways distal to the TM or about the nature of outflow itself, such as segmentalization, may be at play. Regarding segmentalization, for example, success and failure of MIGS may be influenced by where in the eye (superior, inferior, nasal, or temporal) the surgery is performed.

To better elucidate the complexities of AHO, this report describes the development of aqueous angiography as a proof-of-concept method for visualizing AHO *in situ*. Aqueous angiography is a real-time AHO imaging technique performed under physiologic IOP. Aqueous angiography is a form of anterior segment angiography similar to iris angiography or scleral angiography, but focuses on aqueous outflow as opposed to blood flow.[32, 33] By introducing the tracer into the anterior chamber, aqueous angiography provides simultaneous information 360 degrees circumferential around the limbus and does not exclude contributions from any portion of the AHO pathways. Unfortunately, none of the current AHO imaging methods [8–25] are concurrently live, real-time, physiologic, simultaneously circumferential, or allow for contributions from the entire AHO pathways. This report uses aqueous angiography to demonstrate and study segmental AHO in the enucleated model eye. Aqueous angiography serves as a technique to better understand the anatomy and physiology of AHO in the eye. Aqueous angiography may further act as a tool to guide and improve glaucoma surgeries for IOP lowering and vision preservation in the future.

## Methods

### Aqueous Angiography

Non-pigmented pig eyes (n = 46) were obtained from abattoirs (Visiontech, Texas; USDA approved for processing meat for human consumption; 3–5 years old; shipped on blue ice) within 24 hours of death. Right and left pig eyes might not have necessarily come from the same animals due to the lack of indication of animal number. Human eyes (n = 6) were obtained from San Diego Eye Bank within 48 hours of death. Eyes were trimmed of extraocular tissue, orientated by inferior oblique insertion location,[34] and pinned to styrofoam. A Lewicky AC maintainer (BVI Visitec, United Kingdom) was inserted through a 1 mm sideport into the anterior chamber. Balanced salt solution (BSS; Alcon, Texas) was introduced for a 1 hour pre-perfusion period at room-temperature (RT) with a reservoir height set at 5 or 16 inches above the eye to provide a gravity-delivered pressure of 10 or 30 mm Hg. This timing was determined by previous literature [35] employing a ~30 minute pre-perfusion period in pigs for steady state prior to data acquisition and preliminary experiments (n = 7) where the weight of BSS delivered to pig eyes at 10 or 30 mm Hg was monitored and plotted over time demonstrating a linear relationship for at least 30 minutes prior to the 1 hour time point (r^2^>0.994; coefficient of variance = 0.0045). Eyes were kept moist with RT BSS soaked gauze. Simultaneously, 25% fluorescein (Akorn, Illinois) was diluted at RT in BSS to 2.5%. This concentration was chosen as 2.5% fluorescein has been described for clinical use in live humans as an intraocular capsular stain [36]. Prior to imaging, aqueous humor was exchanged with the 2.5% fluorescein in BSS with a reservoir height set for 10 or 30 mm Hg. Alternatively, 3 kD fluorescent dextrans [37, 38] (Life Technologies, California; diluted to 2.5 mg/ml in BSS) were used (n = 3) at 10 mm Hg in pig eyes.

Aqueous angiography was performed with a Spectralis HRA+OCT (Heidelberg Engineering, Heidelberg, Germany; excitation wavelength: 486 nm, transmission filter set at > 500 nm). Eyes were placed in front of the device and imaged with a 55 degree lens using a 25 diopter focus. Confocal scanning laser ophthalmoscopic (cSLO) infrared images were taken to center the eye. Prior to fluorescein application, cSLO fluorescent angiography images were taken to provide a standard pre-tracer intensity background image which appeared black. Subsequent images were taken at various time points in different cardinal positions or face-on. To prevent image signal intensity saturation over time, the laser sensitivity setting on the Spectralis was adjusted with each image to set the central fluorescent signal in the anterior chamber to just under signal saturation. Videos were alternatively taken.

### Aqueous Angiography Image Processing and Statistics

**Fig 1:**
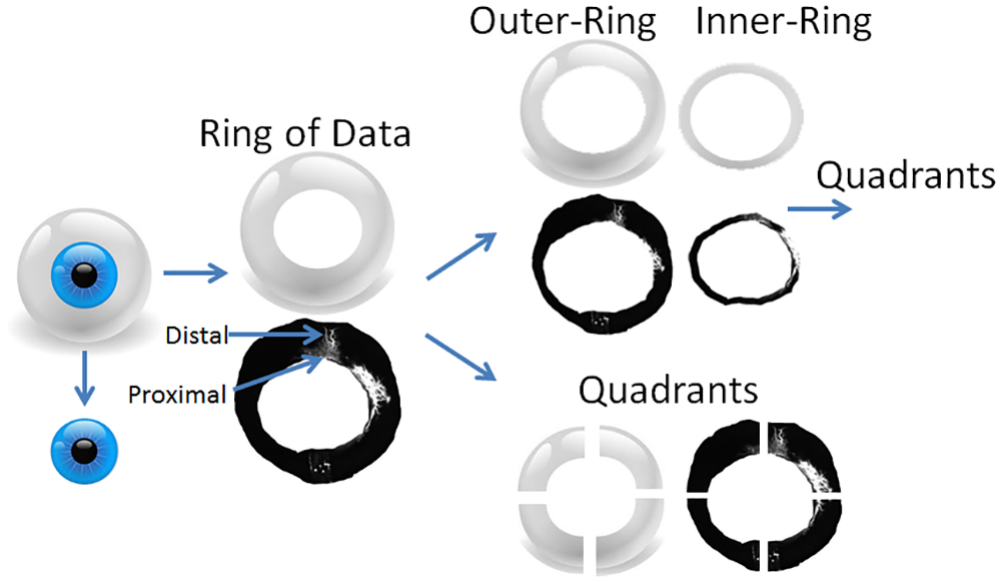
Image Processing for Quantitative Analyses. The cornea/limbal border and the globe horizon were manually segmented with the anterior chamber and any information beyond the globe horizon removed to create a total ring of angiographic data. While this ring certainly compressed the information due to the globe curvature, this view allowed for the entire angiographic image to be seen 360 degrees around the limbus due to the available lenses on the Spectralis. The segmented cornea/limbal border was then identified and expanded to 115% in Photoshop (transform selection tool) to separate an inner- versus outer-ring approximating more proximal from distal signal. Qualitatively, a 115% enlargement encompassed the peri-limbal signal of the TM/Aqueous plexus (AP)/CC complex. The a) total, b) inner-, and c) outer-rings could then be divided into equal quadrants (superior-nasal [SN], superior-temporal [ST], inferior-nasal [IN], and inferior-temporal [IT]).

Aqueous angiographic images were opened in Photoshop CS5 (v.12x32) for image processing and pixel intensity measurements. Angiographic signal within the anterior chamber and beyond the globe horizon for each image was cropped out to create a total ring of angiographic data to allow for quadrant analyses (Fig 1). While face-on images compressed the angiographic data due to the globe curvature, this was the only view that allowed 360 degrees simultaneous imaging circumferential around the limbus. Alternatively, creation of anterior segment flat-mounts by removal of the anterior segment with placement of relaxing incisions could minimize globe curvature distortion, but then this approach would require sampling of angiographic images over time from different eyes effectively negating the key advantage of real-time imaging seen in aqueous angiography.

Quadrants from the ring were created based on location (Fig 1; superior-temporal [ST], superior-nasal [SN], inferior-temporal [IT], inferior-nasal [IN]). Quadrants could be organized based on relative rank signal intensity (highest to lowest) or locational identity (ST/SN/IT/IN). Total angiographic rings were also separated into inner- and outer- rings by expanding the internal border of the total ring by 115% to approximate a division between proximal AHO regions (TM/Aqueous plexus [AP]/CC complex) from more distal (Fig 1). Qualitatively, a 115% enlargement encompassed the peri-limbal signal of the TM/Aqueous plexus (AP)/CC complex. Also, using a similar image analyses approach from human surgical images during glaucoma surgery where a lighted probe was inserted into SC, a 115% enlargement was confirmed to include TM/SC with the lighted probe serving as a marker (data not shown). Both inner- and outer-rings were then divided into quadrants as well.

For quantitative analyses, the background fluorescein angiography signal was first established by determining “average background pixel intensity” from a region of interest in the center of each eye from the pre-tracer image mentioned above. Average pixel intensities for all rings/quadrants were then obtained and background adjusted by subtracting out the “average background pixel intensity.” To control for manual adjustments to the Spectralis laser sensitivity settings during image acquisition, the background adjusted average pixel intensity from each quadrant/ring was divided by the numerical value on the Spectralis laser sensitivity setting to yield a normalized intensity value. Relative aqueous angiography signal per quadrant (%-values; Tables 1 and 2) was calculated by dividing the normalized intensity values of each quadrant by that of the corresponding ring.

**Table 1:** Relative Aqueous Angiography in Different Quadrants Stratified by Signal Intensity in the Pig Eye.

**10 mm Hg Total Data**	**Mean (%)**	**SD**	**SEM**	**30 mm Hg Total Data**	**Mean (%)**	**SD**	**SEM**
Highest (1)	54.8	13.4	3.2	Highest (1)	53.1	13.8	3.3
(2)	27.3	8.5	2.0	(2)	26.2	7.9	1.9
(3)	12.6	5.3	1.3	(3)	14.7	8.6	2.0
Lowest (4)	5.3	5.5	1.3	Lowest (4)	6.0	5.5	1.3
**10 mm Hg Inner-Ring**	**Mean (%)**	**SD**	**SEM**	**30 mm Hg Inner-Ring**	**Mean (%)**	**SD**	**SEM**
Highest (1)	54.2	14.9	3.5	Highest (1)	48.6	15.3	3.6
(2)	27.1	9.7	2.3	(2)	25.6	7.4	1.7
(3)	13.0	7.7	1.8	(3)	17.5	6.3	1.5
Lowest (4)	5.7	5.9	1.4	Lowest (4)	8.1	6.6	1.6
**10 mm Hg Outer-Ring**	**Mean (%)**	**SD**	**SEM**	**30 mm Hg Outer-Ring**	**Mean (%)**	**SD**	**SEM**
Highest (1)	65.2	18.6	4.4	Highest (1)	53.6	13.4	3.2
(2)	20.7	9.6	2.3	(2)	26.9	10.1	2.4
(3)	9.0	7.6	1.8	(3)	12.4	6.0	1.4
Lowest (4)	5.1	5.5	1.3	Lowest (4)	7.1	6.0	1.4

The mean relative signal intensities (%) were determined, irrespective of locational identity, and ranked from 1–4 (highest to lowest). Clear differences between the quadrants (highest to lowest) supported segmental and non-uniform outflow in this *ex vivo* system. SD = standard deviation, SEM = standard error of the mean.

**Table 2:** Relative Aqueous Angiography in Different Quadrants Stratified by Location in the Pig Eye.

**10 mm Hg Total Data**	**Mean (%)**	**SD**	**SEM**	**30 mm Hg Total Data**	**Mean (%)**	**SD**	**SEM**
ST	26.5	21.4	5.0	ST	21.5	17.4	4.1
IT	27.5	16.9	4.0	IT	24.0	20.1	4.7
IN	28.3	25.9	6.1	IN	21.3	17.7	4.2
SN	17.1	18.4	4.3	SN	33.3	23.7	5.6
**10 mm Hg Inner-Ring**	**Mean (%)**	**SD**	**SEM**	**30 mm Hg Inner-Ring**	**Mean (%)**	**SD**	**SEM**
ST	28.5	22.2	5.2	ST	24.5	15.8	3.7
IT	30.8	20.9	4.9	IT	27.9	16.7	3.9
IN	26.1	22.0	5.2	IN	21.9	16.8	4.0
SN	14.6	17.0	4.1	SN	25.6	20.7	4.9
**10 mm Hg Outer-Ring**	**Mean (%)**	**SD**	**SEM**	**30 mm Hg Outer-Ring**	**Mean (%)**	**SD**	**SEM**
ST	26.8	26.6	6.3	ST	24.8	19.1	4.5
IT	25.4	24.7	5.8	IT	18.3	14.6	3.4
IN	23.0	29.2	6.9	IN	21.6	20.0	4.7
SN	24.8	27.7	6.5	SN	35.4	24.0	5.7

ST = superior-temporal, IT = inferior-temporal, IN = inferior-nasal, SN = superior-nasal. SD = standard deviation, SEM = standard error of the mean.

To test for (A) segmentalization at 10 and 30 mm Hg, a Kruskal-Wallis test (SAS, version9.3; SAS Institute, Inc, Cary, NC) was performed on the mean difference among quadrants determined by relative rank of signal intensity (highest to lowest). To test (B) whether a single best or worst quadrant of outflow by location existed, another Kruskal-Wallis test was performed on the mean difference among quadrants defined by location (ST/IT/SN/IN). Kruskal-Wallis test is a non-parametric statistical test used to detect mean differences of rank values across groups. It was chosen in this analyses because of relative small sample size which does not have enough statistical power to check the normal distribution assumption used in ANOVA, a commonly used statistical test in this situation. In both tests for (A) and (B), the null hypothesis is that there is no difference in the mean outflow among all four quadrants. By rejecting the null hypothesis of (A), it indicates that there is an overall statistically significant difference in mean signal intensity among four ranked quadrants, or there is at least one quadrant with statistically significant mean difference from the rest quadrants. On the other hand, by rejecting the null hypothesis of (B), it indicates that there is an overall statistically significant difference in mean signal intensity among four quadrants defined by location (ST/IT/SN/IN), or there is at least one quadrant with statistically significant mean difference from the rest quadrants. The failure to reject either null hypothesis would mean that there was a statistically significant difference in the mean signal intensity among all four quadrants, or there exist a best or worst quadrant by rank (A) or by location (B) or that simply more data is needed.

Concordance between proximal and distal regions meant that the quadrant with greatest signal intensity in the inner-ring matched the location of the quadrant with the greatest signal in the outer-ring (for example, both ST). To look for inner- and outer-ring quadrant concordance, the number of concordant eyes were counted and the percentage of concordant eyes was compared to the probability of binomial distribution being 25% based on a binomial distribution test of proportion. The 25% was chosen because this was the expected percentage of concordance (4 concordant combinations among 16 possible combinations between a pair of 4 quadrants– 4 inner- and 4 outer-rings).

### Optical Coherence Tomography (OCT)

Anterior segment OCT [39–42] (n = 3) was concurrently conducted in some instances of aqueous angiography in pig to determine if angiographically positive regions would show vessel anatomy compatible for AHO. The anterior segment module (Heidelberg Engineering, Germany) on Scleral Mode was used. Single line scans with a 15 degree scan angle (3.9 micrometer axial and 11 micrometer lateral resolution; ~ 4.5 mm) were taken with oversampling (ART = 20) in angiographically positive/negative regions.

### Histological Processing and Microscopy

After aqueous angiography with fluorescent dextrans for ~2 min in pig eyes, the intracameral fluorescent dextran solution was exchanged with 4% paraformaldehyde (PFA) for 10 minutes at 10 mm Hg. Anterior caps including the TM and cornea were then removed and placed in PFA for another 15 minutes. Wedges including the angle were cut from angiographically positive and negative regions and placed in Tissue-tek (Sakura, Japan). Ten micron cryosections were cut onto Superfrost Plus slides (VWR), air dried, washed in PBS, mounted with DAPI, and viewed under a Keyence BZ-X700 digital imaging microscope (Keyence, Illinois). Sections were imaged using a 4x plan-fluor lens with a 0.13 numerical aperture. All images were taken using identical settings for illumination and image capture sensitivity (Keyence imaging software v.1.51). For FITC-dextrans (EX BP 470/30, DM 495, EM BP 520/35) and DAPI (EX BP 360/40, DM 400, EM BP 460/50) appropriate filters were used respectively. For fluorescent quantitative analyses, after removal of non-specific Descemet’s membrane signal, average fluorescence pixel intensity was determined in a region of interest centered on the angle (Photoshop CS5 v.12x32) from which background signal in each slide (determined by sampling empty AC) was subtracted to obtain a background adjusted intensity value. Statistical comparison of the background adjusted intensity value was conducted with unpaired two-sample Student’s t-test. Human eyes were similarly processed for paraffin sectioning, hematoxylin-eosin (H&E) staining, and light microscopy.

## Results

### Aqueous Angiography in Pig

**Fig 2:**
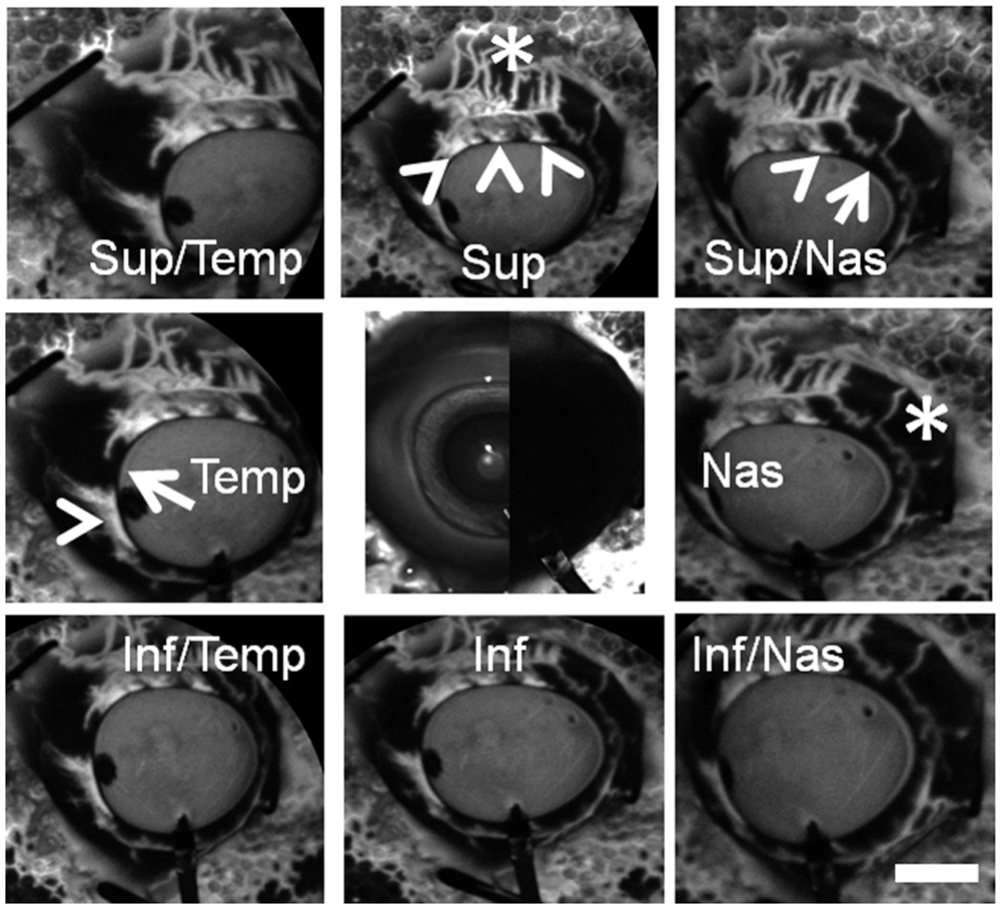
Aqueous Angiography Shows Segmental Patterns. Images from nine cardinal positions were taken on a representative pig eye demonstrating segmental and differentially emphasized angiographic patterns. Arrowheads denoted regions of peri-limbal proximal (TM/AP/CC) signal and asterisks highlighted regions of distal signal (episcleral). Arrows showed areas of relatively low per-limbal signal. The central image was a composite image of cSLO infrared (left side) and pre-injection background (right side) images. Note that the pre-injection background was even less intense than that of the stained styrofoam (polygonal background pattern) that the eye was attached to. Sup = superior; temp = temporal, nas = nasal; inf = interior. Scale bar = 1 cm.

Aqueous angiography yielded excellent images with good signal-to-noise ratio and a dark background (Fig 2). Different regions of the eye clearly demonstrated areas of angiographic positivity near the limbus in a segmental pattern (Fig 2; arrowheads) separated by areas of relatively less peri-limbal angiographic signal (Fig 2; arrows). In species such as pig, a well-defined SC is replaced by a similar but more interwoven structure named the aqueous plexus (AP).[10, 43] Thus, peri-limbal angiographically positive areas most likely represented a proximal outflow pathway TM/AP/CC complex. Distally, these complexes could be seen leading into aqueous and episcleral veins (Fig 2; asterisks) although these two vein types could not be accurately distinguished from each other here.

**Fig 3:**
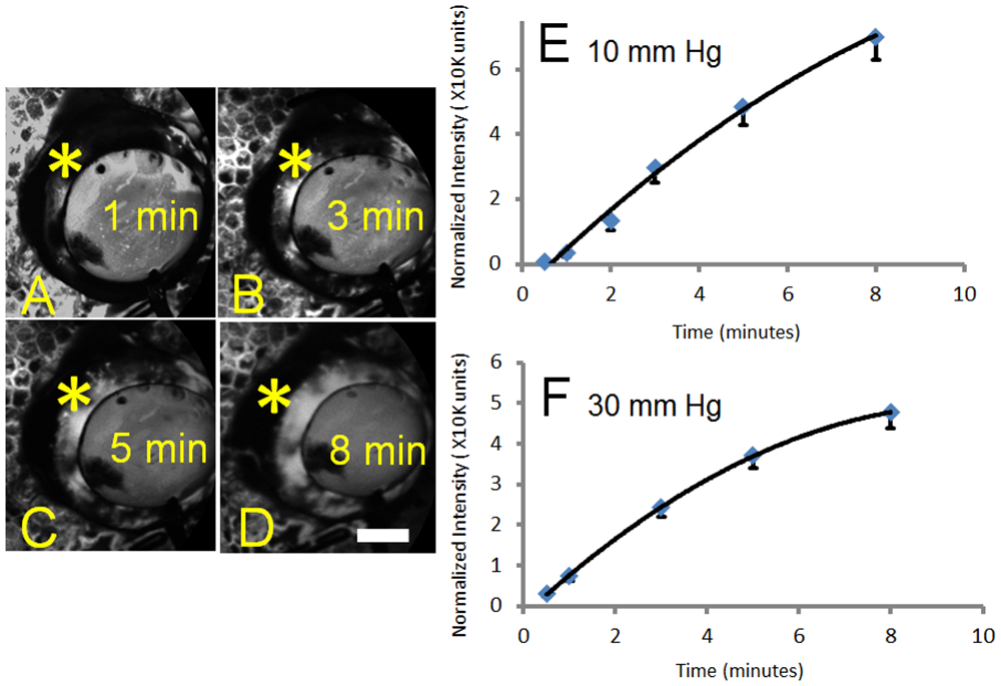
Aqueous Angiography Signal Intensity Strengthens and Plateaus with Time. (A-D) Aqueous angiography signal (asterisks) increased in one eye with 2.5% fluorescein over time from 1 to 8 minutes. (E) Total normalized pixel intensity values from 18 total eyes were recorded as a function of time with perfusion at 10 mm Hg. (F) Total normalized pixel intensity values from 18 total eyes were recorded as a function of time with perfusion at 30 mm Hg. Graphs showed mean +/- standard error. Given that eyes were enucleated, episcleral veins eventually ended with nowhere for the fluorescein to go other than to accumulate on the surface, possibly explaining the plateau. This accumulation appeared slower at 10 mm Hg compared to 30 mm Hg. As such, segmental and relative differences in angiographic signal in different parts of the eye were more readily observed at earlier time points. Scale bar = 1 cm.

Face-on aqueous angiography images were taken from 18 eyes (9 right and 9 left) at either 10 or 30 mm Hg (total 36 eyes). With time, aqueous angiographic signal increased and started to plateau qualitatively and quantitatively (Fig 3). Signal plateau (slower at 10 mm Hg than 30 mm Hg) may have been due to collection of fluorescein on the ocular surface at severed or opened episcleral veins. Thus, the 2 minute and 1 minute time points were used for experiments done at 10 and 30 mm Hg respectively for subsequent quantitative and statistical analyses given their positions on still relatively linear portions of the curves (Fig 3E/3F).

**Fig 4:**
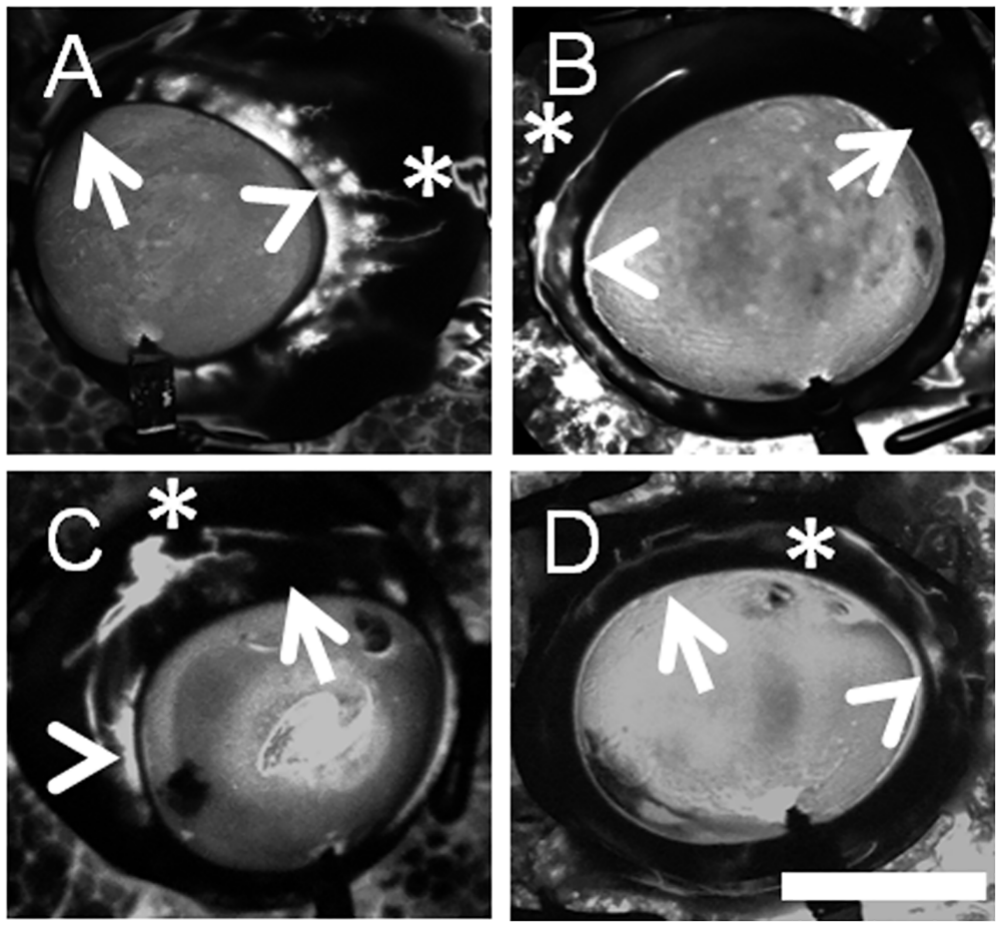
Aqueous Angiography Shows Different Patterns in Different Pig Eyes. Robust segmental angiographic signal was seen with regions of proximal (TM/AP/CC; arrowheads), distal (asterisks), and relatively diminished (arrows) regions. (A/B) Different eyes showed different patterns where in some cases, more proximal angiographic signal (arrowheads) was located near adjacent to more distal angiographic signal (asterisks). (C/D) In other eyes, more proximal angiographic signal (arrowheads) traveled distally to different locations in the eye (asterisks). Scale bar = 1 cm.

Qualitatively, proximal and distal angiographic signals could be seen exhibiting variable patterns (Fig 4). For example, proximally, angiographically positive (Fig 4; arrowheads) and negative (Fig 4; arrows) regions could be distinguished. Distally, signal was also apparent (Fig 4; asterisks). While sometimes proximal signal was located near and adjacent to distal signal (Fig 4A/4B arrowheads and asterisks), other times, proximal signal was found linked to but in a separate region as distal signal (Fig 4C/4D arrowheads and asterisks).

Quantitatively, while face-on images compressed the angiographic data due to globe curvature, this approach allowed for simultaneous appreciation of all four quadrants of the eye (Fig 1). From these views, images were processed (removal of the anterior chamber signal and any data beyond the globe horizon) to create a “ring” of angiographic information that was then divided into quadrants (ST/IT/SN/IN). The ring was further divided into an inner-ring (proximal signal) versus an outer-ring (distal signal).

To test for segmentalization, a Kruskal-Wallis test was performed on relative outflow (%) information at 10 and 30 mm Hg with quadrants stratified by rank (lowest to highest) regardless of location to detect an overall difference in the relative signal intensities (and thus segmentalization) among the quadrants (Table 1). In all cases ([10 mm Hg: total data, inner- and outer-ring] and [30 mm Hg; total data, inner- and outer-ring]) an overall difference was found (*p*<0.001) suggesting segmental patterns.

To test whether a single best or worst quadrant by location existed, another Kruskal-Wallis test was performed on relative signal at 10 and 30 mm Hg with quadrants stratified by location to search for a mean difference (either higher or lower) among the quadrants (Table 2). This time, in no case was a statistically significant overall difference found ([10 mm Hg: total data *p* = 0.24; inner-ring *p* = 0.06; outer ring *p* = 0.16] and [30 mm Hg: total data *p* = 0.33; inner-ring *p* = 0.47; outer ring *p* = 0.86]) suggesting that neither an overall best nor worst quadrant for signal intensity by location was detected.

Lastly, concordance (meaning the best proximal and distal outflow quadrants by location matched) could not be detected. At 10 mm Hg, 7/18 eyes were found to have identical best inner- and outer-ring quadrants (38.9%, 95% confidence intervals = 17.3–64.3%; *p* = 0.17), demonstrating a lack of concordance. At 30 mm Hg, 3/18 eyes were found to have matching best inner- and outer-ring quadrants (16.7%, 95% confidence intervals = 3.6–41.4%; *p* = 0.41), also demonstrating a lack of concordance.

### Aqueous Angiography Represents Aqueous Humor Outflow (AHO)

To test whether angiographic signal was located in AHO pathway lumens, as opposed to representing surface conjunctival staining, optical coherence tomography (OCT) and fluorescent dextran experiments were performed.

**Fig 5:**
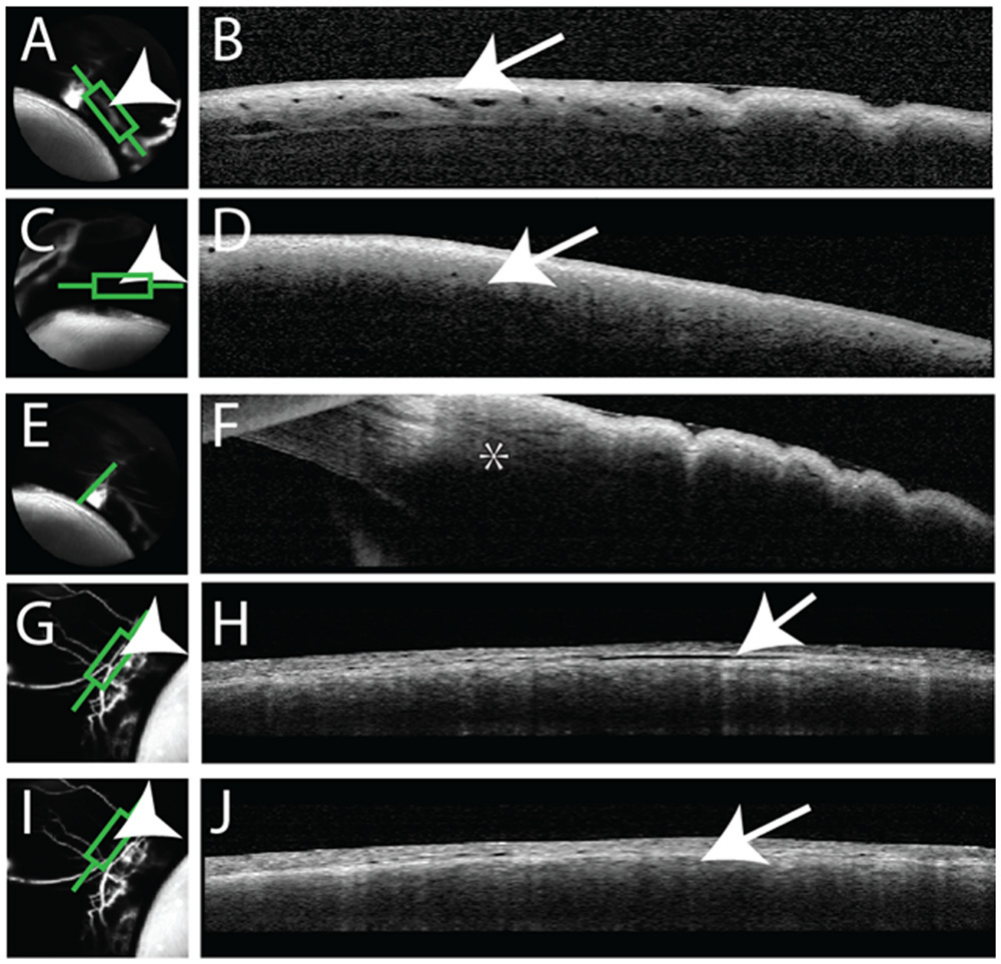
Aqueous Angiography and Optical Coherence Tomography (OCT). Aqueous angiography was conducted in pig eyes in parallel with anterior segment OCT. (A/G) Angiographically positive areas (arrowheads) correlated with (B/H) intrascleral lumens on OCT (arrows). (C/I) In contrast, angiographically lacking areas (arrowheads) were (D/J) devoid of intrascleral lumens on OCT (arrows). (E) Angiographically positive areas could be associated with a classical “side-ways Y”[39] aqueous vein (asterisk).

While conducting aqueous angiography with 2.5% fluorescein at 10 mm Hg, OCT was simultaneously conducted (n = 3) on angiographically positive and negative areas with an anterior segment lens. In angiographically positive areas (Fig 5A/5G), OCT showed multiple intrascleral lumens (Fig 5B/5H) reminiscent of AHO pathways. OCT performed in angiographically negative areas (Fig 5C/5I) were devoid of lumens (Fig 5D/5J). In some cases (Fig 5E/5F), a classic “sideways-Y”[39] aqueous outflow structure could be seen in association with angiographically positive areas.

**Fig 6:**
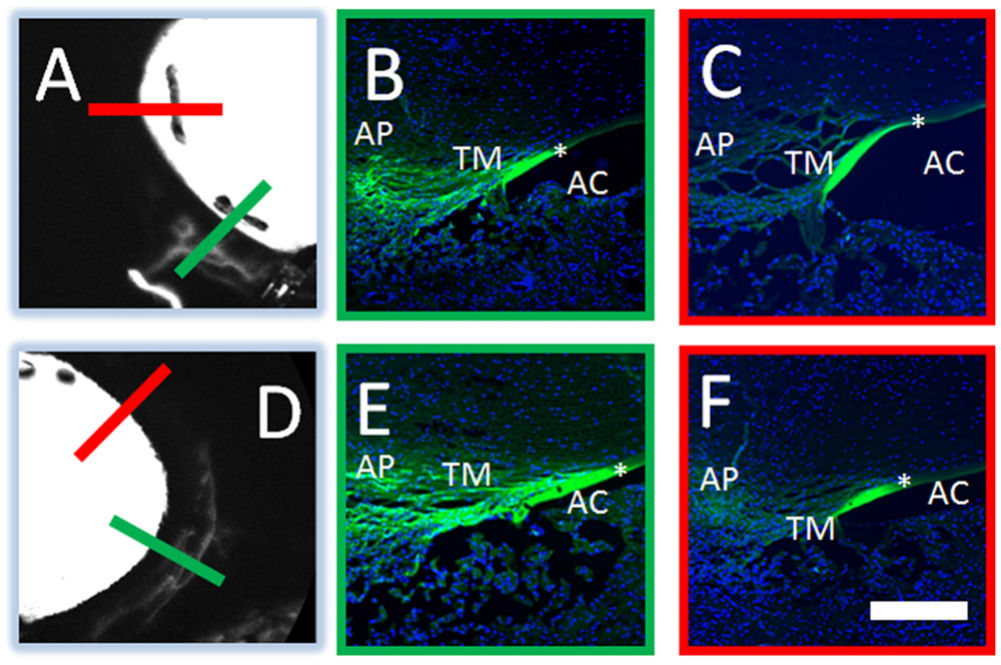
Aqueous Angiography Localizes to AHO Pathways. Aqueous angiography was conducted with 3 kD fixable fluorescent dextrans in pig eyes. Two representative eyes (A-C and D-F) are shown here. Angiographically positive (A/D; green lines) or diminished (A/D; red lines) regions were identified with aqueous angiography and then sectioned. In the first eye (A-C), angiographically positive (green line in A corresponds to panel B) but not angiographically negative (red line in A corresponds to panel C) regions showed trapping of dextrans within outflow pathways. In the second eye (D-F), angiographically positive (green line in D corresponds to panel E) but not angiographically negative (red line in D corresponds to panel F) regions also showed trapping of dextrans within outflow pathways. Note similar degree of non-specific fluorescence seen in strips of Descemet Membrane in all cases (asterisks). AP = aqueous plexus, TM = trabecular meshwork, AC = anterior chamber. Scale bar = 100 microns.

To follow aqueous angiography at a microscopic level, 3 kD fixable and fluorescent dextrans were used in substitution of free fluorescein (n = 3). These small molecular weight dextrans were fluorescent and fixable due to attached fluorescein and lysine moieties respectively. After aqueous angiography was performed, positive and negative regions were marked, and the eyes were perfusion fixed to trap fluorescent dextrans to nearby walls of AHO pathway lumens (Fig 6). Frozen sections were then created for fluorescent microscopy. As expected, angiographically positive areas showed greater fluorescent dextran deposition in the angle compared to angiographically negative areas (Fig 6B/6E [green line from A/D] vs. C/F [red line from A/D]). Quantitative comparison of background adjusted intensity values in angiographically positive compared to negative areas showed a statistically significant increase (69.23 ± 3.46 vs. 18.20 ± 2.21 background adjusted intensity units; average ± SEM; n = 12 sections for both conditions; *p*<0.001; two-tailed Student’s *t*-test).

### Aqueous Angiography in Human

**Fig 7:**
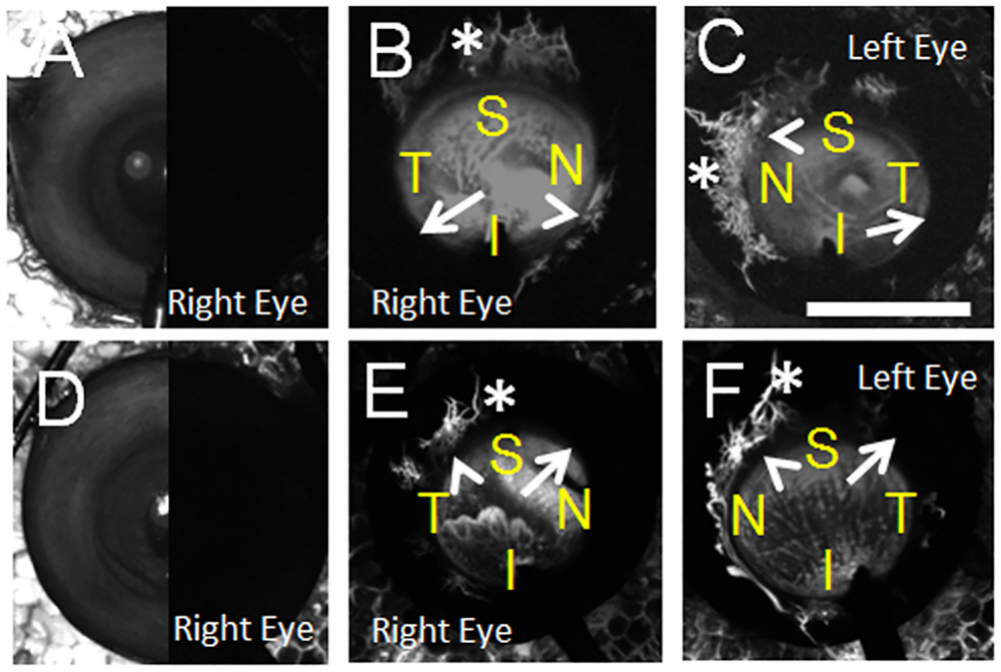
Aqueous Angiography in Enucleated Human Eyes. Aqueous angiography was performed on enucleated eyes from two female subjects not known to have glaucoma at 10 mm Hg (subject 1 = (A-C) and subject 2 = (D-F)). Both right and left eyes from each subject were investigated and shown at 10–25 seconds. (A/D) Composite cSLO infrared (left-side) and pre-injection background images (right-side) are shown from the right eyes of these two subjects. S = superior; T = temporal, N = nasal; I = inferior. AC = anterior chamber, TM = trabecular meshwork, SC = Schlemm’s Canal. Scale bars = 1 cm.

**Fig 8:**
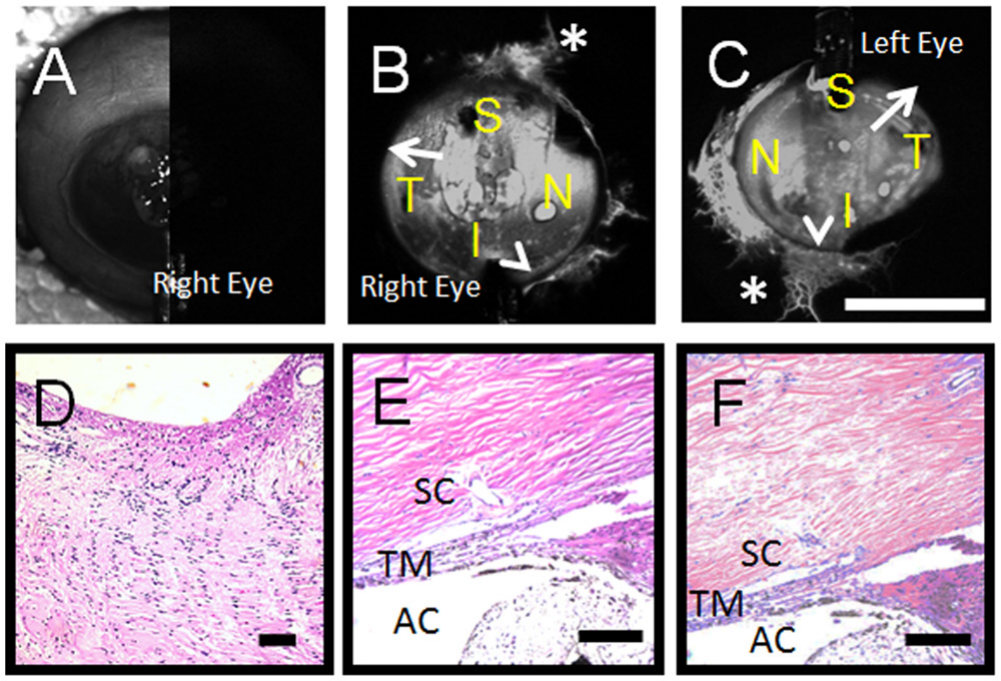
Aqueous Angiography and Histology in Enucleated Human Eyes. Aqueous angiography was performed on enucleated eyes from one male subject at 30 mm Hg (A-C). (A) Composite cSLO infrared (left-side) and pre-injection background images (right-side) are shown from the right eye only. (D) This subject was not known to have glaucoma and the optic nerve of the right eye showed lack of cupping on paraffin sectioning. Also in the right eye, angle structures were grossly similar at the light microscopic level comparing regions of greater (E; S region of panel B) or lesser (F; ST region of panel (C) angiographic signal. S = superior; T = temporal, N = nasal; I = inferior. AC = anterior chamber, TM = trabecular meshwork, SC = Schlemm’s Canal. Scale bars = 100 micron (black) and 1 cm (white).

Aqueous angiography was performed on 3 pairs of enucleated human subject eyes at 10 mm Hg (79 year-old and 83 year-old females who passed away from cardiopulmonary arrest; Fig 7A–7C and 7D–7F) and 30 mm Hg (81 year-old male who passed away from leukemia; Fig 8A–8C) all not known to have glaucoma. Aqueous angiography was performed as in pig. Segmental patterns were observed with regions of positive peri-limbal (Figs 7 and 8; arrowheads), lack of peri-limbal (Figs 7 and 8; arrows), and distal (Figs 7 and 8; asterisks) regions of angiographic signal. Comparing the two female subjects investigated at 10 mm Hg, different patterns could be seen comparing the individuals (Fig 7B vs. 7E and 7C vs. 7F) and between the eyes of a single individual (Fig 7B vs. 7C and 7E vs. 7F). In the 81 year-old male, paraffin sections through the right optic nerve did not demonstrate glaucomatous changes (Fig 8D). At the light microscopic level for the 81 year-old male, no obvious differences could be distinguished between regions of relatively better (Fig 8E; right eye S) or worse (Fig 8F; right eye ST) angiographic signal. Video of the 79 year-old female’s left eye showed real-time relative changes (S1 Video).

## Discussion

Aqueous angiography yielded high quality images that were related to and usable in the study of AHO. Use of a 486 nm laser to specifically excite fluorescein allowed for high signal-to-noise background ratio images with backgrounds essentially devoid of fluorescence. While similar anatomy might have been seen using white-light and filters, the level of detail seen with aqueous angiography likely could not be achieved. This level of image clarity allows aqueous angiography to be a proof-of-concept method in model eyes to simulate what visualizing AHO could look like *in vivo*.

In this model system, aqueous angiography clearly demonstrated segmental angiographic patterns in both pig and human eyes qualitatively and in pig eyes quantitatively. Segmental angiographic patterns were confirmed statistically by assessing overall non-uniformity across quadrants stratified by *rank intensity*. Despite segmentalization, no overall differences were found among the quadrants stratified by *location* implying the lack of a best quadrant of AHO. Additionally, in each individual eye, the region of best proximal signal did not necessarily correlate (show concordance) with the region of heaviest distal signal. As such, identifying the location of the best distal episcleral draining vein would not necessarily elucidate which quadrant of the proximal outflow pathway fed this episcleral vein. One limitation to this data was that the quantitative results were obtained on pig eyes. Future experiments are planned in other species to determine generalizability.

Together, these results have potential clinical implications. For example, with segmental patterns, success of trabecular bypass MIGS may not be equal with placement anywhere in the eye but rather in specific regions. Additionally, without a best draining quadrant identified, MIGS should not always be placed in one assumed location. In other words, typical nasal MIGS placement may explain its variable success. Also, if concordance could not be detected between the best flowing proximal and distal outflow regions, surface evaluation of the eye [24] prior to surgery for the best appearing or blanching episcleral vein with or without irrigation/aspiration may not always be sufficient to guide surgery.[44] Complete outflow information is needed for the best surgical outcomes.

The best outflow imaging technique needs to be a combination of live, real-time, physiologic, simultaneously circumferential, and not exclude contributions from any point of the AHO pathways. Previous methods using microbeads were not real-time requiring enucleation, fixation, sectioning, and microscopy. Channelography and canalography also are not real-time and only focus on distal outflow (by injecting the tracer post-TM) essentially implying incorrectly that the TM has no contribution to AHO.[20–24] Moreover, channelography and canalography do not always provide simultaneously circumferential information. In both cases, fluorescein (sometimes mixed with viscoelastic) is deposited in SC through a canaloplasty probe while it is being pulled out of the eye. In this way, a “train” of information can be obtained as the probe sweeps through precluding simultaneous 360 degree appreciation. Channelography and canalography are further non-physiologic as they involve tracer introduction at unknown and likely high pressure through injection. For irrigation/aspiration methods, an irrigation/aspiration unit is placed in the angle to push fluid at high pressure out of the eye to visualize blanching downstream episcleral veins.[44] Here, lack of physiologic conditions is the limitation. Even if studying outflow at high pressure was acceptable, the lack of concordance between proximal and distal flow shown herein, albeit in pig eyes, limits this methodology for MIGS guidance.

Aqueous angiography is a real-time and physiologic outflow imaging technique that allows for acquisition of simultaneous 360 degree circumferential information without excluding the TM. However, a significant limitation of aqueous angiography in this report is the use of enucleated eyes. Because of this, temporal implications needed to be considered. In enucleated eyes, with time, aqueous angiography signal increased and started to plateau (Fig 3). This might have been expected since in an enucleated eye AHO ultimately ends on the ocular surface at severed or open episcleral veins. As fluorescein could not return back into the body, it likely accumulated. This result may have given a false appearance of greater uniformity of signal with time. This is why early time points, that more greatly emphasized and imaged segmental patterns, were used for quantitative analyses. Additional limitations with using enucleated eyes include post-mortem disruption with cellular viability changes, segmental findings possibly resulting from episcleral venous blood clotting, or perfusion induced tissue swelling all of which could be better addressed using *in vivo* studies.

Future research will be dedicated to testing additional tracers, conducting a finer histological/biochemical comparison between areas of greater or lesser signal, and attempting aqueous angiography in live organisms. Testing additional tracers will be important as any angiographic method is limited by the unique (both pro and con) characteristics of each tracer. It is possible that different patterns may emerge with different tracers that may have different excitation/emission profiles, molecular weight, protein binding, or etc. Here we chose fluorescein because its molecular weight is close to water (compared to a microbead) and also because fluorescein has been described for intraocular use.[36] Also, while no obvious difference between greater or lesser outflow regions were seen in the human angle at the level of light microscopy, we suspect that this was the case due to the inherent limitations of light microscopy and the requirement for more detailed studies (such as electron microscopy [45]) to even touch upon differences between normal and glaucomatous tissues. However, with an emerging method to distinguish regions of greater or lesser angiographic intensity, future studies can be done. Lastly, adapting this method for live animals will require a unique and balanced organization between a perfusion rig (with pumps/motors/micro-manipulators etc.) and the Spectralis which is a human clinical device. While difficult, this will allow for improved studies where, for example, temperature and a more delicate exchange of aqueous humor and tracer through the sulcus could occur.

For humans, future plans include bringing aqueous angiography into the operating room to visualize outflow in live normal and glaucomatous individuals. The methods in this report were developed to be suitable for this purpose. Gravity delivery of intraocular fluid through a Lewicky AC maintainer [46] is a common surgical practice. The use of 2.5% fluorescein diluted in balanced salt solution is described by the American Academy of Ophthalmology as a capsular stain.[36] The remaining challenge will be to appropriately situate an angiographer in a sterile fashion over a patient in the operating room.

Once applied in humans, aqueous angiography may be used to improve MIGS results. One approach may be to place the MIGS where the signal is initially greatest since the surgeon knows that the anatomy is supportive of outflow there. Alternatively, the surgeon could also place the MIGS toward the regions of poorest signal to attempt to recruit underutilized areas. Either way, customizing and guiding MIGS placement in individual glaucoma patients is a powerful concept rooted in improving glaucoma surgeries based on fundamental understandings of the eye. In conclusion, aqueous angiography represents an exciting new tool for future anterior segment AHO research. Aqueous angiography may also have potential in humans to improve surgical glaucoma treatment.

## Supporting Information

S1 Video Aqueous Angiography in an Enucleated Human Left Eye. Aqueous angiography was conducted on the left eye of subject 1 (Fig 7C). The AC maintainer entered the eye inferior at 6 o’clock. Nasal was 9 o’clock and temporal was 3 o’clock. Note that there was positive nasal aqueous angiographic signal at 9 o’clock at 1 second. The nasal region increased in angiographic signal from 1–4 seconds with the start of angiographic signal superior-temporal at 5 seconds. Angiographic signal started to appear inferior-temporal at 10 seconds. Temporally, no angiographic signal was seen until two discrete points arose at nearly 30 seconds.
(MP4)
